# Claudin-2 deficiency reveals a corticomedullary calcium gradient driving kidney stone formation

**DOI:** 10.1172/JCI201055

**Published:** 2025-12-01

**Authors:** Benjamin D. Humphreys

Kidney stones are common, affecting 15%–20% of the population over their lifetimes, with recurrence rates approaching 50% (1). The most common form is composed of calcium oxalate and initiates as Randall’s plaques — hydroxyapatite crystals in the medullary interstitium. These crystals grow and in time rupture into the urinary space, serving as a nidus for calcium oxalate stone growth (2). The major risk factor for calcium oxalate kidney stone formation is hypercalciuria. Claudin-2 is a paracellular iron channel that mediates most of the calcium absorption in the kidney and is expressed primarily in the proximal tube, loop of Henle, and intestinal epithelium. Prior work characterizing mice with global claudin-2 knockout (*Cldn2*-KO mice) revealed lifetime hypercalciuria, intestinal calcium hyperabsorption, and development of nephrocalcinosis resembling Randall’s plaques (3). The *Cldn2*-KO mouse thus modeled the early steps in human idiopathic hypercalciuria and calcium oxalate stone formation.

With the aim of dissociating the effects of *Cldn2* knockout in both kidney and intestine, in this issue of the *JCI*, Behm et al. report on the creation of mice with tubule-specific constitutive deletion using a *Pax8*-cre driver and inducible tubule-specific *Cldn2* deletion using *Pax8*-rtTA (4). Mice with kidney tubule–specific *Cldn2* deletion experienced only transient hypercalciuria just after birth, followed by normalization. Similarly, adult-onset tubule-specific deletion of *Cldn2* also produced only transient hypercalciuria. Tubule-specific *Cldn2*-KO mice compensated with mildly increased parathyroid hormone because of negative calcium balance and upregulated expression of distal tubule calcium transporters, presumably secondary to increased distal delivery of calcium. Despite lifetime normocalciuria, these mice still developed inner medullary nephrocalcinosis at 6–12 months of age.

Clinically, individuals who form idiopathic hypercalciuric kidney stones have defects in proximal tubule calcium reabsorption, and the “vas washdown” hypothesis holds that distal delivery of luminal calcium leads to increased interstitial calcium concentration in the inner medulla and papilla as a consequence of vasa recta blood flow and countercurrent exchange (similar to handling of sodium chloride and urea) (5). Direct evidence supporting the vas washdown hypothesis has been lacking. The discovery that kidney tubule–specific *Cldn2*-KO mice developed nephrocalcinosis despite normocalciuria led Behm and colleagues to test whether the interstitial calcium gradient was increasing in these mice. The authors identified a corticomedullary calcium gradient that was highest in papilla and exacerbated in tubule-specific *Cldn2*-KO mice, providing a mechanistic explanation for the development of nephrocalcinosis and support for the vas washdown hypothesis (4).

These studies clearly implicate interstitial medullary and papillary calcium accumulation — rather than hypercalciuria per se — as being critical in the early pathogenesis of calcium oxalate stone formation. The finding makes sense given that hypercalciuria in isolation is insufficient to drive stone formation (6, 7). These results also reorient therapeutic approaches for the prevention of kidney stones to target this mechanism and provide a new model to test such therapies.

## Version 1 12/01/2025

Electronic publication
